# Peripheral neuropathy as a very rare symptom in a patient with Niemann–Pick type C with negative enzymatic evaluation: a case report

**DOI:** 10.1186/s13256-021-03136-2

**Published:** 2022-01-12

**Authors:** Mohammad Barzegar, Fatemeh Valaee, Shadi Ghoreishizadeh

**Affiliations:** 1 Pediatric Health Research Center, Tabriz University of Medical Sciences, Tabriz, Iran; 2 Razi Hospital, Tabriz University of Medical sciences, Tabriz, Iran

**Keywords:** Niemann–Pick, Niemann–Pick type C, Peripheral neuropathy, Mutation

## Abstract

**Background:**

Niemann–Pick is a rare metabolic disease distinguished by lysosomal storage defects. This disease is characterized by sphingomyelinase acid deficiency, causing its accumulation in various organs such as the kidneys, spleen, liver, brain, and nerves. Niemann–Pick disease is categorized into four groups: A, B, C, and D. Peripheral neuropathy is an extremely rare complication in patients with Niemann–Pick type C, which certainly leads to neurologic deterioration.

**Case presentation:**

We report a case of Niemann–Pick type C disease in a 3-year-old Iranian Azeri female patient who was hospitalized twice. The first time was at 1 month of age with symptoms of splenomegaly, jaundice, and elevated liver enzymes, and the second time was at around age 2 for loss of mental and physical abilities. The patient presented with failure to thrive. According to paraclinical examinations, mildly delayed myelination along with a nonspecific periventricular hypersignal intensity was seen. Interestingly, the patient’s Niemann–Pick type C enzymatic function was evaluated twice and was negative on both occasions, while she was positive for *NPC1* and *NPC2* gene examinations.

**Conclusions:**

In this study, despite the enzymatic study being negative, Niemann–Pick type C disease was finally confirmed, revealing the importance of mutations in Niemann–Pick type C pathogenesis. Besides, peripheral neuropathy was diagnosed in this patient as a very rare symptom of Niemann–Pick type C.

## Introduction

Niemann–Pick (NP) is a rare, inherited, heterogeneous disease that is characterized by a group of lysosomal fat storage dysfunctions affecting the body’s ability to metabolize fat. Based on molecular and biochemical tests and criteria, this rare disease includes two subclasses, as follows: accumulation of sphingomyelin inside the cell due to sphingomyelinase deficiency is the first subclass of NP disease composed of types A and B, while excessive storage of free cholesterol in endosomal or lysosomal compartments is known as the second subclass of Niemann–Pick disease, comprising types C and D [1].

Niemann–Pick type C (NPC) is an autosomal recessive disease resulting from defective lipid storage at an incidence of 1:120,000. Since NPC is mostly known as an inherited disease, having a sibling would be a reliable predictor for this disease. The age at which clinical manifestations are reported and the rate of neurological symptom progression are extremely variable, yet nonspecific symptoms delay the diagnosis of the disease, and it has even been shown to be diagnosed 5 years after the onset of neurological manifestations [1, 2]. In these patients, administration of miglustat as soon as possible can reduce neurological symptoms and result in improved therapeutic outcomes [3]. Neuropathologically, its main feature is axonal spheroid formation in the central nervous system; however, loss of neurons and accumulation of intracranial cytoplasmic compounds should not be ignored. Peripheral neuropathy is a very rare complication in patients with NPC. In this regard, there are a small number of cases revealing this complication in literature. It has only been reported in patients who were under miglustat medication, a synthetic analog of d-glucose that acts as a competitive agent and reversible inhibitor of the glucosylceramide synthase enzyme [4, 5]. Approaches that reduce the gap between disease onset and diagnosis would be vital for patients.

We describe a case of NPC1 in a 42-month-old girl who was first hospitalized at 1 month of age with splenomegaly and jaundice. After 4 months, her liver tests and skin color returned to normal. However, she gradually lost motor speech skills and was hospitalized again with a similar complaint at about 30 months old. The patient was referred to the neurology ward because of neurologic symptoms, mainly motor and cognition regression and intermittent dystonic posture aggravated with passive and active movements.

## Case presentation

### Clinical examinations

The patient was a 42-month-old Iranian Azeri girl with family history of asymptomatic splenomegaly in her maternal grandmother and aunt. She had a birth weight of 2600 g with head circumference of 34 cm.

The infant had a dark color owing to cholestasis and direct hyperbilirubinemia. At about 1 month of age, she was hospitalized for 4 days because of prolonged jaundice, splenomegaly, and high liver enzymes. Then, she was treated with phenobarbital because of prolonged cholestasis along with possible biliary atresia, and at 3 months the patient’s skin color and liver tests were normal. The patient had a developmental delay so that she started walking at approximately 18 months, and at the age of 2 she started to speak a few words. Around the age of 2, she was being visited by a physician and underwent physiotherapy because of gait and balance disturbance. Gradually, she lost previously learned skills including motor and speech information, being hospitalized at around the age of 2.5 years. The patient’s orientation showed an appreciable change in her level of awareness such that she could not recognize her parents. Besides, the patient was suffering from generalized postural dystonia and could not follow simple instructions. There were no symptoms of dysmorphic features. Her head circumference was 44 cm. Her eye movements were normal. An enlarged spleen without nodules was noted, causing abdominal tenderness. Deep tendon reflexes were absent. The child had speech and language impairments such that she could not put two words together. She could only say up to ten words. The child was also found to be suffering from failure to thrive (FTT) condition.

### Paraclinical tests

**Fig. 1:**
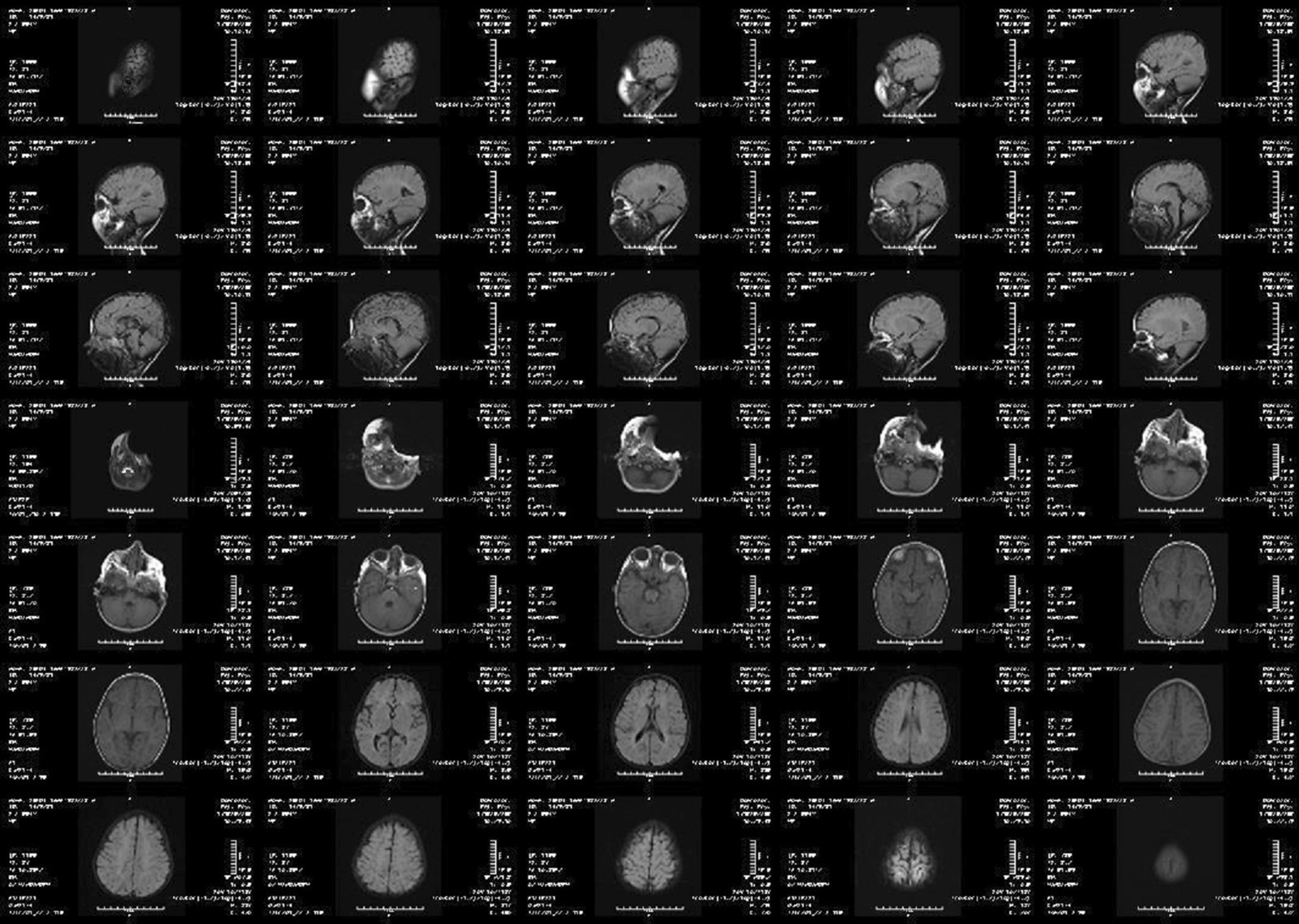
Brain MRI scan shows that all parameters were normal except mildly delayed myelination and nonspecific periventricular hyper-signal intensity

The newborn screening filter paper test was normal. The patient had a normal complete blood count (CBC) with differential. Lactate and ammonia were also normal. A complete newborn screening test is summarized in Table 1. Electromyography (EMG) and nerve conduction velocity (NCV) showed motor-sensory neuropathy (MSN), especially motor-sensory axonal neuropathy (MSAN). Brain MRI was performed at 30 months of age with the following results: cerebrum and cerebellum exhibited normal cortical sulcation; no abnormalities seen in the basal ganglia, internal capsule and corpus callosum, or thalamus; brain stem, sella, and pituitary were normal. There were only two notable findings, one of which was a mildly delayed myelination and the second of which was a nonspecific periventricular hypersignal intensity (Fig. 1). The patient was then directed to bone marrow aspiration (BMA) regarding hematologist counseling, in which NPC enzyme activity and amount were normal. Interestingly, the patient’s NPC enzymatic function was evaluated twice and was negative both times. At 36 months, she lost the remaining motor skills, along with severe dystonia and a moderate degree of spasticity and encephalopathy. Therefore, an electroencephalogram (EEG) was taken and the results showed diffuse delta activity with an encephalopathic pattern. At that appointment, the patient was lethargic and sometimes fell asleep following laughter, indicating cataplexy. Finally, the patient was referred to the diagnostic genetic center for *NPC1* and *NPC2* gene testing, which was positive. The patient is currently under miglustat q.d. and baclofen b.i.d.

**Table 1:** Newborn screening tests

Newborn screening tests	Results	Normal range	Unit
Neonatal screening			
AST	75	26–75	U/L
ALT	27	11–46	U/L
ALP	178	25–500	U/L
Bill	7.5	3–17	µmol/L
PT	12	11–14	Seconds
PTT	30	25–40	Seconds
Chol	192	158.8 ± 44	mg/dL
TG	180	136.9 ± 97	mg/dL
Serum ammonia	25	15–45	m/dL
Serum lactate	1/5	0.5–2.2	mmol/L
Hgb	Unremarkable	13.7–20.1	g/dL
TSH	< 10	< 10	mU/L
17-OH progesterone	< 15	< 15	ng/dL
Galactose	< 15	< 15	mg/dL
Galactose 1-P-uridyl transferase	> 20	> 20	%Activity
Biotinidase	< 5	< 5	%Activity
Succinylacetone	> 30	> 30	µmol/L
Disorders of amino acid metabolism			
Arginosuccinase	0.5	< 1.5	µmol/L
Leucine + isoleucine	113	< 300	µmol/L
Valine	70	< 250	µmol/L
Citruline	8	< 60	µmol/L
Methionine	19	8–100	µmol/L
Phenylalanine	29	< 150	µmol/L
Tyrosine	36	< 125	µmol/L
Disorders of beta oxidation of fatty acids			
MCADD, VLCADD, LCHADD	Unremarkable	–	–
Disorders of carnitine metabolism			
Acyl carnitines	Unremarkable	–	–
Disorders of organic acids			
Isovalerylcarnitine	Unremarkable	–	–
Glutaric acid	Unremarkable	–	–
Defects of urea cycles			
Citrulline	Unremarkable	–	–
Argininosuccinate	Unremarkable	–	–
Acid sphingomyelinase deficiency			
Acid sphingomyelinase^a^	449	200–3500	pmol/spct 20 h
Beta-galactosidase deficiency			
Beta-galactosidase^a^	542.5	200–500	nmol/spct 21 h
Acid β-glucosidase (Gaucher Disease)			
Acid β-glucosidase^a^	0.79	0.5–3.5	pmol/spct 20 h

*AST* Aspartate aminotransferase; *ALT* Alanine aminotransferase; *ALP* Alkaline phosphatase; *Bill* Bilirubin; *PT* prothrombin time; *PTT* Partial thromboplastin time; *Chol* Cholesterol; *TG* Triglyceride; *Hgb* Hemoglobin; *TSH* Thyroid-stimulating hormone; *MCADD* Medium-chain acyl-CoA dehydrogenase deficiency; *VLCADD* Very-long-chain acyl-CoA dehydrogenase deficiency; *LCHADD* Long-chain 3-hydroxy acyl-CoA dehydrogenase

^a^Lysosomal enzymes from dried blood

## Discussion

The patient underwent a complete clinical and paraclinical examination, and the diagnosis of Niemann–Peak disease was confirmed after the onset and progression of symptoms. Notably, the patient’s enzymatic activity for NPC was reported negative twice. Peripheral neuropathy was eventually diagnosed in this patient as a very, very rare symptom in patients with Niemann–Peak disease. In approximately 20–40% of NPC patients, the age of onset of neurological symptoms is in the range of 2–6 years, consistent with this report [6, 7].

In line with our study, a previous study revealed that hepatosplenomegaly together with cholestatic liver disease were predominant symptoms in infants with NPC [8]. Here, we obtained two negative results from the enzymatic assay in a patient with NPC, which may be because of the normal function of sphingomyelinase. However, if the systemic and neurological symptoms predict higher risk of NPC, mutations in *NPC1* and *NPC2* genes should be examined, not only for documentation but also for carrier recognition and prenatal diagnosis. In this regard, genotype–phenotype studies have shown a strong association between frameshift or nonsense mutations with neurological manifestations in NPC1 patients [9].

Having more information about NPC and accurately determining its pathogenesis could lead to early diagnosis, which is critical for patients. Early diagnosis of the disease followed by administration of miglustat, a competitive inhibitor of glucosylceramide synthase, can delay the onset of neurological symptoms and prolong survival [10].

## Conclusion

This case report of NPC with peripheral neuropathy is the first in Iran’s medical literature. In conclusion, it is recommended that infants with systemic symptoms such as unexplained splenomegaly with or without hepatomegaly, continuous cholestatic jaundice, fetal hydrops, and a high score on the NPC Suspicion Index tool should be referred to a genetic laboratory for possible NPC diagnosis. Also, in this study, despite the enzymatic study and its negative results twice, NPC disease was finally confirmed, showing that mutations in NPC genes should also be considered. Peripheral neuropathy was also diagnosed in this patient as a very rare symptom of NPC, the exact mechanisms of which should be determined in further studies.

## Data Availability

All are available if required.

## References

[1] Vanier MT. Niemann–pick diseases. Handb Clin Neurol. 2013;113:1717–21. 10.1016/B978-0-444-59565-2.00041-1 23622394

[2] Usui M, Miyauchi A, Nakano Y, Nakamura S, Jimbo E, Itamura S, Adachi K, Nanba E, Narita A, Yamagata T. Miglustat therapy in a case of early-infantile Niemann-Pick type C. Brain Develop. 2017;39:886–90. 10.1016/j.braindev.2017.05.006 28587793

[3] Lyseng-Williamson KA. Miglustat: a review of its use in Niemann-Pick disease type C. Drugs. 2014;74:61–74. 10.1007/s40265-013-0164-6 24338084

[4] Rosenbaum AI, Maxfield FR. Niemann-Pick type C disease: molecular mechanisms and potential therapeutic approaches. J Neurochem. 2011;116:789–95. 10.1111/j.1471-4159.2010.06976.x PMC3008286 20807315

[5] Iodice R, Dubbioso R, Topa A, Ruggiero L, Pisciotta C, Esposito M, Tozza S, Santoro L, Manganelli F. Electrophysiological characterization of adult-onset Niemann-Pick type C disease. J Neurol Sci. 2015;348:262–5. 10.1016/j.jns.2014.12.005 25537619

[6] Zech M, Nübling G, Castrop F, Jochim A, Schulte EC, Mollenhauer B, Lichtner P, Peters A, Gieger C, Marquardt T. Niemann-Pick C disease gene mutations and age-related neurodegenerative disorders. PloS One. 2013;8:e82879. 10.1371/journal.pone.0082879 PMC3875432 24386122

[7] Yerushalmi B, Sokol RJ, Narkewicz MR, Smith D, Ashmead JW, Wenger DA. Niemann-pick disease type C in neonatal cholestasis at a North American Center. J Pediatr Gastroenterol Nutr. 2002;35:44–50. 10.1097/00005176-200207000-00011 12142809

[8] Gotti G, Marseglia A, De Giacomo C, Iascone M, Sonzogni A, D’Antiga L. Neonatal jaundice with splenomegaly: not a common pick. Fetal Pediatr Pathol. 2016;35:108–11. 10.3109/15513815.2015.1130762 26847548

[9] Spiegel R, Raas-Rothschild A, Reish O, Regev M, Meiner V, Bargal R, Sury V, Meir K, Nadjari M, Hermann G. The clinical spectrum of fetal Niemann-Pick type C. Am J Med Genet A. 2009;149:446–50. 10.1002/ajmg.a.32642 19206179

[10] Patterson MC, Vecchio D, Prady H, Abel L, Wraith JE. Miglustat for treatment of Niemann-Pick C disease: a randomised controlled study. Lancet Neurol. 2007;6:765–72. 10.1016/S1474-4422(07)70194-1 17689147

